# Analgesic effects of medicinal plants and phytochemicals on chemotherapy‐induced neuropathic pain through glial modulation

**DOI:** 10.1002/prp2.819

**Published:** 2021-10-22

**Authors:** Ji Hwan Lee, Nari Kim, Sangwon Park, Sun Kwang Kim

**Affiliations:** ^1^ Department of Physiology College of Korean Medicine Kyung Hee University Seoul Korea; ^2^ Department of Science in Korean Medicine Graduate School Kyung Hee University Seoul Korea; ^3^ Department of Korean Medicine Graduate School Kyung Hee University Seoul Korea

**Keywords:** analgesia, chemotherapy‐induced peripheral neuropathy, glia, medicinal plant, phytochemical

## Abstract

Chemotherapy‐induced peripheral neuropathy (CIPN) frequently occurs in cancer patients. This side effect lowers the quality of life of patients and may cause the patients to abandon chemotherapy. Several medications (e.g., duloxetine and gabapentin) are recommended as remedies to treat CIPN; however, usage of these drugs is limited because of low efficacy or side effects such as dizziness, nausea, somnolence, and vomiting. From ancient East Asia, the decoction of medicinal herbal formulas or single herbs have been used to treat pain and could serve as alternative therapeutic option. Recently, the analgesic potency of medicinal plants and their phytochemicals on CIPN has been reported, and a majority of their effects have been shown to be mediated by glial modulation. In this review, we summarize the analgesic efficacy of medicinal plants and their phytochemicals, and discuss their possible mechanisms focusing on glial modulation in animal studies.

## Graphical abstract

Medicinal plants and phytochemicals alleviate CIPN by modulating spinal glial activity.

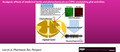

**Abbreviations**
CIPN: Chemotherapy‐induced peripheral neuropathy
CNS: central nervous system
DPK: Divya‐peedantak‐Kwath
GBT: Gyejigachulbu‐tang
GJG: Goshajinkigan
KSOT: Kei‐kyoh‐zoh‐soh‐oh‐shin‐bu‐toh
NYT: Ninjin'yoeito
PEA: *N*‐Palmitoylethanolamine
QoL: quality of life
WLT: Wen‐luo‐tong

## 1 INTRODUCTION

Chemotherapy is a standard first‐line therapy for various types of cancer.^1^, ^2^, ^3^ However, chemotherapeutic agents, such as oxaliplatin, paclitaxel and vincristine, are neurotoxic.^4^, ^5^, ^6^ Due to this, more than 60% of chemotherapy‐treated patients suffer from peripheral sensory symptoms approximately 24–48 h after chemotherapy infusion.^7^, ^8^ Abnormal sensory symptoms after chemotherapy manifest as cold and mechanical allodynia in a glove‐and‐stocking distribution.^9^ Such neuropathic pain that occurs after chemotherapy lowers the quality of life (QoL) of patients and is one of the primary reasons why patients abandon chemotherapy.^10^, ^11^, ^12^, ^13^ To treat these side effects, antidepressant or anticonvulsant drugs, such as duloxetine or gabapentin, are referred for patients as a first‐line treatment; however, these drugs have been reported to be accompanied by dizziness, nausea, somnolence and vomiting.^14^, ^15^, ^16^, ^17^

Plants have long been used not just as food, but also as drugs, since ancient times (e.g., using the willow tree for pain killers). Salicylic acid, the major phytochemical of willow bark, was synthesized by science in modern times. This development opened up the possibility that natural compounds could have benefits that can be applied to mankind faster and more precisely. Therefore, a quarter of the currently‐consumed drugs originate from plants.^18^ Traditional medicine in East Asia including China, Japan and Korea contains various types of natural products for health. Thousands of years ago, “Breaking the code of damage from cold” (Korean name: Sang Han Lon) summarized and organized the prescriptions of natural products for drugs. Whole plants or disassembled plants (e.g., bark, berry, leaf, flower and root) were treated as a paste or decoction (water or hydroalcoholic). These medicinal herbs were administered either alone or as a composite formula. Medicinal herbs have been prescribed for the treatment of colds or various types of pain. Recent research has revealed that medicinal herbs have various pharmacological activities, such as antimicrobial, antioxidant, anti‐inflammatory, and anti‐nociceptive activities.^19^ The potency of medicinal herbs is mediated by their bioactive compounds and phytochemicals.^20^, ^21^, ^22^

Glial activation is commonly observed in various animal models of pain, such as inflammatory pain and neuropathic pain models.^23^, ^24^ Astrocytes and microglia in the central nervous system (CNS) respond to noxious stimulation or are activated in pathological pain conditions.^24^, ^25^, ^26^ Upon the induction of pain, they change their morphologies: astrocytes show hypertrophy with thick branches; ameboid shape of soma and short and thick processes with hypertrophy appear in microglia.^27^, ^28^, ^29^ Similarly, chemotherapeutic agents elicit microglial activation, followed by activation of astrocytes.^30^ With their conformational changes, gliotransmitters, which act on nearby neurons and glia themselves, are released from activated glia.^31^ Cytokines, well‐known gliotransmitters, are also released as signaling molecules^32^ and sensitize neurons, resulting in pain.^33^ For example, IL‐1β facilitates phosphorylation of ERK and NMDA receptors, and TNF‐α strengthens excitatory postsynaptic currents.^34^, ^35^

In the past few years, our group has elucidated the potency of medicinal herbs and their phytochemicals on chemotherapy‐induced peripheral neuropathy (CIPN) using animal models.^36^, ^37^, ^38^, ^39^ We have shown the hypertrophy of glial cells in CIPN models and the analgesic effect of medicinal herbs and their phytochemicals through inhibition of glial activation.^36^, ^37^, ^38^ According to the data reported by us and others, medicinal herbs and their phytochemicals are applicable to suppress CIPN, and these studies suggest that glia could be an efficient therapeutic target. Thus, in this review, we summarize and discuss the recent findings regarding the analgesic effects of medicinal herbs and phytochemicals and their glial modulation in CIPN animals (Table 1).

**TABLE 1:** Efficacy of medicinal herbs and phytochemicals on CIPN animal model via glial modulation

Types of treatment	Chemotherapy (dosing/strain)	Types of glial cell	Findings: behavioral changes	References
			Findings: changes of glia and its action	
Gyejigachulbu‐tang (p.o., 200, 400, 600 mg/kg, 5 times)	Oxaliplatin (i.p., 6 mg/kg, single, SD rat)	Astrocyte, Microglia (Spinal Cord)	Cold hyperalgesia ↓ Mechanical hyperalgesia ↓	Ahn et al. (2014)^36^
			# of GFAP positive cell ↓ # of OX‐42 positive cell ↓	
Kei‐kyoh‐zoh‐soh‐oh‐shin‐bu‐toh (p.o., 0.3, 1.0 g/kg, daily)	Oxaliplatin (i.p., 36 mg/kg, 9 times, C57BL/6)	Astrocyte (Spinal Cord)	Mechanical allodynia ↓	Andoh et al. (2019)^43^
			# of GFAP positive cell ↓	
Wen‐luo‐tong (not mentioned in the original article)	Oxaliplatin (i.p., 36 mg/kg, 9 times, Wistar rats)	Astrocyte (Spinal Cord)	Mechanical allodynia ↓ Mechanical hyperalgesia ↓	Deng et al. (2016)^45^
			# of GFAP positive cell, IOD ↓ Substance P and TNF‐α mRNA ↓	
Aconiti Tuber (p.o., 300 mg/kg, 5 times)	Oxaliplatin (i.p., 6 mg/kg, single, SD Rat)	Astrocyte, Microglia (Spinal Cord)	Cold allodynia ↓ Mechanical allodynia ↓	Jung et al. (2017)^37^
			# of GFAP positive cell ↓ TNF‐α, IL‐1β ↓	
Aqueous extract of Lithospermi Radix (p.o., 250 mg/kg, 24 times)	Oxaliplatin (i.p., 10 mg/kg, 2 times, C57BL/6)	Astrocyte, Microglia (Spinal Cord)	Mechanical hyperalgesia ↓	Cho et al. (2016)^60^
			# of GFAP positive cell ↓ # of Iba‐1 positive cell ↓ # of TNF‐α positive cell ↓	
Astragali Radix (p.o., 300 mg/kg, 21 times or 28 times)	Oxaliplatin (i.p., 36 mg/kg, 15 times, SD rat; 12 mg/kg, 8 times, Pirc rat)	Astrocyte, Microglia (Spinal Cord, Brain)	Cold hyperalgesia ↓ Mechanical allodynia ↓ Mechanical hyperalgesia ↓	Ghelardini et al. (2017)^51^
			# of GFAP positive cell ↓ # of GFAP positive cell ↓ @ Cg, S1, M1, PAG, mfb # of Iba‐1 positive cell ↓ @ S1, M1, PAG, mfb	
*Cinnamomum cassia* (p.o., 100, 200, 400 mg/kg, 5 times)	Oxaliplatin (i.p., 6 mg/kg, single, SD rat)	Astrocyte, Microglia (Spinal Cord)	Cold allodynia ↓ Mechanical allodynia ↓	Kim et al. (2016)^38^
			# of GFAP positive cell ↓ # of Iba‐1 positive cell ↓	
Evodiae Fructus (p.o., 200 mg/kg, 5 times)	Oxaliplatin (i.p., 6 mg/kg, single, SD rat)	Astrocyte, Microglia (Spinal Cord)	Cold allodynia ↓	Kim et al. (2013)^54^
			# of GFAP positive cell, GFAP density ↓ # of OX‐42 positive cell, OX‐42 density ↓	
*Vitis vinifera* 60% ethanol extract (p.o., 300 mg/kg, 15 times) in vitro (50 μg/ml, 4 h incubation)	Oxaliplatin (i.p. 36 mg/kg, 15 times, SD rat) in vitro (100 μM, 4 h)	Primary Cultured Astrocyte, Astrocyte (Spinal Cord)	Mechanical allodynia ↓ Mechanical hyperalgesia ↓ Cold hyperalgesia ↓	Micheli et al. (2018)^67^
			# of GFAP positive cell ↓ (in vitro) TBARS basal level ↓	
Icariin (p.o., 25, 50, 100 mg/kg, 8 times)	Paclitaxel (i.p., 24 mg/kg, 3 times, SD rat)	Astrocyte, Microglia (Spinal Cord)	Mechanical allodynia ↓	Gui et al. (2018)^73^
			# of GFAP positive cell ↓ NF‐κB (p65) phosphorylation ↓ TNF‐α, IL‐1β, IL‐6↓ SIRT1 ↑ H4‐K16Ac ↓	
Melatonin (i.p., 20 mg/kg, single)	Oxaliplatin (i.p., 20 mg/kg, 4 times, SD Rat)	Astrocyte (Spinal Cord)	Mechanical allodynia ↓ Heat hyperalgesia ↓	Wang et al. (2017)^77^
			GFAP expression ↓ TNF‐α, IL‐1β, MCP‐1 MIP‐1α mRNA ↓	
*N*‐Palmitoylethanolamine (i.p., 30 mg/kg, 20 times)	Oxaliplatin (i.p., 36 mg/kg, 15 times, SD Rat)	Astrocyte, Microglia (Spinal Cord, Brain)	Cold allodynia ↓	Mannelli et al. (2015)^81^
			# of GFAP positive cell ↓ @ SCDH, S1 # of Iba‐1 positive cell ↓ @ S1 NS neuron response change (Onset ↑, Duration of excitation & Evoked frequency↓)	
Rosmarinic Acid (p.o., 25, 50 mg/kg, 28 times)	Oxaliplatin (i.p., 36 mg/kg, 9 times, SD rat)	Astrocyte (Spinal Cord)	Cold allodynia ↓ Cold hyperalgesia ↓ Mechanical allodynia ↓ Mechanical hyperalgesia ↓	Areti et al. (2018)^87^
			# of GFAP positive cell ↓ TNF‐α and IL‐6 ↓	

Abbreviations: Cg, cingulate cortex; DRG, dorsal root ganglia; GFAP, Glial fibrillary acidic protein; i.p., intraperitoneal; Iba‐1, ionized calcium‐binding adapter molecule 1; IL‐1β, interleukin‐1β; IL‐6, interleukin‐6; IOD, integral optical density; M1, primary motor cortex; MCP‐1, monocyte chemoattractant protein‐1; mfb, medial forebrain bundle; MIP‐1α, macrophage inflammatory protein 1α; NF‐κB, nuclear factor kappa B; NS neuron, nociceptive‐specific neuron; p.o., per os; PAG, periaqueductal grey; Pirc rat, F344/NTac‐Apc^am1137^ rat; S1, primary somatosensory cortex; SCDH, Spinal Dorsal Horn; SD, Sprague‐Dawley; SIRT1, Sirtuin 1, histone deacetylase; TBARS, thiobarbituric acid reactive substances; TNF‐α, tumor necrosis factor‐α; WLR, *Lithospermi* radix extract in hot water.

## 2 DECOCTION OF FORMULAS OF MEDICINAL HERBS OR SINGLE HERBS

### 2.1 Divya‐peedantak‐Kwath

Divya‐peedantak‐Kwath (DPK), which contains 28 medicinal herbs, is a water‐based decoction. Multiple treatments with paclitaxel (total 12 mg/kg, i.p., 6 times) induced heat and mechanical hypersensitivity (hyperalgesia and allodynia) in mice, which were measured by using hot plate (55 ± 0.5℃), tail flick, von Frey and Randall‐Selitto test. Daily oral administration of DPK (69, 205 and 615 mg/kg, 14 times) attenuated paclitaxel‐induced neuropathic pain behaviors in mice. The DPK treatment lowered oxidative stress and inflammation in the peripheral nerves.^40^

### 2.2 Goshajinkigan (Korean name Jesengsingi‐hwan)

Goshajinkigan (GJG) comprised 10 medicinal herbs, including *Cinnamomum cassia* and Aconiti Tuber. Orally treated GJG (1 g/kg, 20 times) inhibited oxaliplatin (total 32 mg/kg, i.p., 8 times)‐induced cold hyperalgesia (cold plate: 4℃) and allodynia (acetone test) from days 3 to 24, and mechanical allodynia (von Frey) from days 11 to 25 in mice. A lower dose of GJG (0.3 g/kg) also had potency on suppressing cold allodynia. The GJG treatment inhibited hyperactivation of Aβ‐ and Aδ‐fibers induced by oxaliplatin.^41^

### 2.3 Gyejigachulbu‐tang

Gyejigachulbu‐tang (GBT) is a decoction of herbal formula, which comprises seven medicinal herbs, including Aconiti Tuber, Evodiae Fructus, and *Zingiber officinale*. In our previous study, multiple oral treatments with GBT (400 and 600 mg/kg, 5 times) in rats suppressed cold (4℃ water) and mechanical (von Frey test) allodynia on the tail induced by a single injection of oxaliplatin (6 mg/kg, i.p.).^36^ Additionally, the GBT treatment suppressed activation of astrocytes and microglia in the spinal dorsal horn and downregulated pro‐inflammatory cytokine (IL‐1β and TNF‐α) levels in the spinal cord after oxaliplatin injection.^36^, ^42^

### 2.4 Kei‐kyoh‐zoh‐soh‐oh‐shin‐bu‐toh

Kei‐kyoh‐zoh‐soh‐oh‐shin‐bu‐toh (KSOT) is a formula of seven medicinal herbs. Repeated oral treatments with KSOT (0.3 and 1 g/kg) showed a relieving effect on oxaliplatin (total 36 mg/kg, 9 times)‐induced mechanical allodynia (von Frey test), but not cold allodynia (acetone test), in mice. Additionally, the KSOT treatment inhibited oxaliplatin‐induced increase in the number of activated spinal astrocytes.^43^

### 2.5 Ninjin'yoeito (Korean name In‐sam‐young‐yang‐tang)

Ninjin'yoeito (NYT) comprises 12 medicinal herbs and contains ginseng as a key ingredient. Daily oral administration of NYT (1 g/kg, 7 times) suppressed oxaliplatin‐induced cold allodynia (acetone test) and mechanical hyperalgesia (von Frey test) in mice. In addition, NYT dose‐dependently reversed oxaliplatin‐induced suppression of neurite outgrowth in primary dorsal root ganglion cells.^44^

### 2.6 Wen‐luo‐tong

Wen‐luo‐tong (WLT) is a formula of four medicinal herbs including *Cinnamomum cassia*. Topical application of WLT to the paws showed efficacy against oxaliplatin (total 36 mg/kg, 9 times)‐induced mechanical allodynia and hyperalgesia (4 and 15 g of von Frey filaments, respectively) in rats.^45^ Moreover, the treatment with WLT had potency against paclitaxel (total 24 mg/kg, 3 times)‐induced mechanical allodynia (von Frey test) from day 6 onward.^46^ On day 31, the WLT treatment inhibited the increase of GFAP and TNF‐α protein and mRNA levels in the rat spinal cord induced by oxaliplatin. The protein level of substance P in the spinal cord was also downregulated in the WLT‐treated group.^45^

### 2.7 Aconiti Tuber (Korean name Buja)

Processed Aconiti Tuber, orally administered (300 mg/kg) for 5 days, had potency on cold (4℃ water) and mechanical (von Frey test) allodynia induced by oxaliplatin (6 mg/kg) on days 3 and 5 in rats.^37^ The treatment with processed Aconiti Tuber (0.27 g/kg for cold, 1 g/kg for mechanical, 8 times) had potency in reducing cold (acetone test) and mechanical (von Frey test) hypersensitivity in mice induced by oxaliplatin (10 mg/kg).^47^ Moreover, the processed Aconiti Tuber (0.3 g/kg) treatment suppressed oxaliplatin (total 32 mg/kg, 8 times)‐induced cold hypersensitivity (acetone test), but not mechanical allodynia (von Frey test) in mice. However, co‐administration of GJG and Aconiti Tuber significantly inhibited mechanical allodynia.^41^ Daily administered Aconiti Tuber (1 g/kg) also effectively lowered paclitaxel (12 mg/kg)‐induced mechanical allodynia (von Frey test) on day 9 in mice.^48^ In our previous study, daily oral administration of processed Aconiti Tuber (300 mg/kg, 5 times) suppressed the activation of astrocytes and downregulated the increase of pro‐inflammatory cytokine (IL‐1β, TNF‐α) levels in the rat spinal cord by oxaliplatin on day 5.^37^

### 2.8 *Acorus calamus* (Korean name Chang‐po)

Two different doses of vincristine (total 0.5 and 0.75 mg/kg, 10 times) elicited heat allodynia (hot plate: 45 ± 0.5℃) and hyperalgesia (hot plate: 52.5 ± 0.5℃) and mechanical allodynia (von Frey test) and hyperalgesia (pin prick test or Randall–Selitto test) in rats. Daily oral treatment with hydroalcoholic (50% ethanol) *Acorus calamus* (100 and 200 mg/kg, 14 times) attenuated vincristine‐induced pain behaviors.^49^, ^50^ The *Acorus calamus* treatment also suppressed TNF‐α levels in peripheral neurons.^50^

### 2.9 Astragali Radix (Korean name Hwang‐gi)

Multiple oxaliplatin injections (total 36 mg/kg, 15 times) induced cold hyperalgesia and mechanical allodynia in rats. Three types of extracts (water, and 20% and 50% ethanol extracts) of Astragali Radix (300 mg/kg, p.o., 21 times) all suppressed mechanical allodynia (von Frey test) and hyperalgesia (paw pressure test). The most effective was the 50% ethanol extract of Astragali Radix, which also suppressed cold allodynia (cold plate: 4 ± 1℃). Furthermore, the 50% ethanol extract inhibited the activation of brain astrocytes and microglia as well as spinal astrocytes.^51^

### 2.10 *Boswellia dalzielii* (Korean name Molyak)

The vincristine rat model (total 1 mg/kg, 10 times) presented heat (hot plate: 51 ± 0.5℃) and mechanical hyperalgesia (Randall‐Selitto test) and cold hyperalgesia (4℃ cold water on the tail). The methanol extract of *Boswellia dalzielii* (500 mg/kg, p.o.) attenuated vincristine‐induced neuropathic pain.^52^

### 2.11 Butea monosperma

Daily treatment with ethanolic extract of *Butea monosperma* leaves (300 and 400 mg/kg, p.o., 14 times) suppressed vincristine (total 0.5 mg/kg, 10 times)‐induced cold (acetone test) and mechanical (von Frey test) allodynia, heat (hot plate test) and mechanical (Randall‐Selitto test) hyperalgesia, and heat hyperalgesia (tail immersion test) in rats, starting on day 3. *Butea monosperma* also showed antioxidant potency in the vincristine model.^53^

### 2.12 *Cinnamomum cassia* (Korean name Yuk‐gye)

Daily oral administration of the water extract of *Cinnamomum cassia* (200 and 400 mg/kg) showed analgesic effects against oxaliplatin (6 mg/kg)‐induced cold allodynia (4℃ water on tail) in rats on days 3 to 5. The oral administration of the water extract of *Cinnamomum cassia* (200 mg/kg, five times) reversed oxaliplatin‐induced spinal glial activations (astrocytes and microglia) and prevented the upregulation of pro‐inflammatory cytokine (IL‐1β and TNF‐α) levels in the spinal cord on day 5.^38^

### 2.13 Evodiae Fructus (Korean name Osuyu)

The water extract of Evodiae Fructus (200 mg/kg), orally administered five times daily, prevented oxaliplatin (6 mg/kg)‐induced cold allodynia (4℃ water on the tail) in rats on days 3 and 5.^54^ The Evodiae Fructus (200 mg/kg, five times) prevented oxaliplatin‐induced activation of spinal astrocytes and microglia, observed on day 5.^54^

### 2.14 *Forsythia viridissima* (Korean name Gae‐na‐ri)

In an intravenously injected oxaliplatin rat model (total 30 mg/kg, 6 times), *Forsythia vidrdissima* (100 mg/kg, p.o., 30 times) administration suppressed mechanical allodynia (von Frey) from day 28. Such treatment with *Forsythia viridissima* recovered the loss of intraepidermal nerve fibers at the footpad by oxaliplatin. In addition, daily treatment with *Forsythia viridissima* (50 mg/kg, p.o.) also showed efficacy in an intraperitoneally injected oxaliplatin mouse model (total 30 mg/kg, 3 times) from day 21 by lowering mechanical hypersensitivity (von Frey).^55^

### 2.15 Gelsemium sempervirens

*Gelsemium sempervirens* (10^−6^, 10^−9^, 10^−18^ dilutions, 11 and 18 times) were intraperitoneally administered in rats. It showed analgesic effects against paclitaxel (total 4 mg/kg, 4 times)‐induced cold (acetone test) and mechanical allodynia (4 g of von Frey), and mechanical hyperalgesia (15 and 26 g of von Frey). Paclitaxel‐induced neuronal loss at the sciatic nerve and plantar skin was restored by treatment with *Gelsemium sempervirens*.^56^

### 2.16 *Ginko biloba* (Korean name Eun‐haeng)

In a vincristine rat model (total 1.2 ml/kg, i.p., 10 times), cold (acetone test) and mechanical (von Frey test) hyperalgesia was attenuated from 15 to 90 min after a single administration of *Ginko biloba* (150 mg/kg, p.o.).^57^

### 2.17 Ginseng (Korean name Insam)

Ginseng is a key component herb of Ninjin'yoeito, which is composed of 12 medicinal herbs. Daily oral administration of ginseng (0.2 g/kg, 7 times) suppressed oxaliplatin (10 mg/kg)‐induced cold allodynia (acetone test) and mechanical hyperalgesia (von Frey test) on day 4.^44^

### 2.18 *Hypericum perforatum* L. (St. John's wort)

A single dose of *Hypericum perforatum* L. (5 mg/kg, i.p.) attenuated oxaliplatin (total 36 mg/kg, 15 times)‐induced cold allodynia (cold plate: 4℃). In addition, *Hypericum perforatum* L. showed efficacy against zalcitabine‐induced neuropathic pain model.^58^

### 2.19 *Lepidium meyenii* (Maca)

Single oral administration of *Lepidium meyenii* (0.5, 1.5, 3, and 10 g/kg) reversed oxaliplatin (total 24 mg/kg, 10 times)‐induced cold hypersensitivity, and at doses of 1.5, 3, and 10 g/kg, showed efficacy against paclitaxel (total 8 mg/kg, 4 times)‐induced cold hypersensitivity (cold plate: 4 ± 1℃) on day 14 in mice. *Lepidium meyenii* treatment also attenuated pain behaviors in an osteoarthritis and nerve damage models.^59^

### 2.20 Lithospermi Radix (Korean name Jacho)

The water extract of Lithospermi Radix (250 mg/kg, p.o., 24 times) reversed oxaliplatin (total 10 mg/kg, 2 times)‐induced mechanical allodynia (4 g of von Frey) in mice from days 14 to 28.^60^ On day 28, the Lithospermi Radix extract suppressed the activation of spinal astrocytes and microglia in the oxaliplatin group. The number of TNF‐α positive cells in the spinal dorsal horn was also decreased by treatment with the Lithospermi Radix extract.^60^

### 2.21 *Salvia miltiorrhiza* (Korean name Dansam)

Multiple administrations of oxaliplatin (total 24 mg/kg, 10 times) induced cold allodynia (cold plate: 4 ± 1℃). It was attenuated by *Salvia miltiorrhiza* (300 and 600 mg/kg, p.o.) on day 14. Interestingly, *Salvia miltiorrhiza* had inhibitory effects in glioblastoma cells, LN‐229.^61^

### 2.22 *Synedrella nodiflora* Gaertn

Eight days after the first paclitaxel (total 10 mg/kg, five times) injection, a single treatment with hydroethanolic extract of *Synedrella nodiflora* (100, 300 and 1000 mg/kg. p.o.) alleviated thermal hyperalgesia (hot plate: 55℃) in rats.^62^ The same amounts of hydroethanolic extract of *Synedrella nodiflora* were administered to rats 5 times daily, and suppressed vincristine (total 0.6 mg/kg, 6 times)‐induced cold allodynia (4℃ water on the tail), mechanical (Randall‐Selitto test) and thermal hyperalgesia (hot plate: 55℃). However, only 100 and 300 mg/kg of *Synedrella nodiflora* had efficacy on tactile allodynia (4 g of von Frey).^63^

### 2.23 *Tanacetum parthenium* (Fiverfew)

Treatment with hydroalcoholic extract of *Tanacetum parthenium* flowers (1000 mg/kg, p.o.) inhibited the reduction of mechanical threshold by oxaliplatin (total 36 mg/kg, 15 times) in rats on day 21. The *Tanacetum parthenium* extract also effectively suppressed pain behaviors in rat models of inflammatory, articular and neuropathic pain.^64^

### 2.24 Tithonia tubaeformis

In the vincristine mouse model (total 1.4 mg/kg, 14 times), a single treatment with hydromethanolic extract of *Tithonia tubaeformis* (100 and 200 mg/kg, p.o.) attenuated mechanical allodynia (von Frey test) and thermal hyperalgesia (52 ± 0.5℃ water on the tail).^65^

### 2.25 Vernonia cinerea

Daily treatment with ethanolic extract of *Vernonica cinerea* leaves (300 and 400 mg/kg, p.o., 14 times) suppressed vincristine (total 0.5 mg/kg, 10 times)‐induced cold (acetone test) and mechanical (von Frey test) allodynia, heat (hotplate: 52.5 ± 0.5℃) and mechanical (Randall‐Selitto test) hyperalgesia in rats from day 3. In addition, the *Vernonica cinerea* extract reverted oxidative tissue damage induced by vincristine.^66^

### 2.26 *Vitis vinifera* (Korean name Podo)

Orally treated *Vitis vinifera* (300 mg/kg, 15 times) prevented mechanical (von Frey test) and cold (4℃ cold plate) hypersensitivity in an oxaliplatin (total 36 mg/kg, 15 times) injected rat model.^67^ The orally treated *Vitis vinifera* potently suppressed the activation of astrocytes and Nrf2 protein expression in the spinal cord on day 21. Moreover, *Vitis vinifera* (50 μg/ml, 4 h incubation) showed antioxidant effect (lowered O_2_^−^ production and lipid peroxidation level) in primary cultures of astrocytes stimulated by oxaliplatin (100 μM).^67^

### 2.27 *Zingiber officinale* (ginger, Korean name Saeng gang)

Single oral treatment with *Zingiber officinale* (100, 300 and 500 mg/kg) reduced oxaliplatin (6 mg/kg)‐induced cold (acetone test) and mechanical (von Frey test) allodynia in mice. The anti‐allodynic effects of *Zingiber officinale* might be mediated by increasing spinal 5‐HT_1A_ receptors.^39^

## 3 PHYTOCHEMICALS

### 3.1 Berberine

Berberine is an alkaloid found in *Berberis vulgaris* and *Berberis croatica* (Korean name Hwang Baek). In a paclitaxel mouse model (total 8 mg/kg, 4 times), intraperitoneally injected Berberine (5, 10, and 20 mg/kg) increased the hot plate latency from 30 to 120 min.^68^

### 3.2 Bergapten

Bergapten is a furanocoumarin found in grapefruit juice, figs, or anise. Intraperitoneally administered bergapten (10 mg/kg, 14 times) showed an analgesic effect on vincristine (total 1 mg/kg, 10 times)‐induced cold (4℃ water on tail), heat (42℃ water on tail) and mechanical allodynia (von Frey test), and mechanical hyperalgesia (paw pressure test) from days 7 to 14. Spinal IL‐1β, TNF‐α, iNOS, COX‐2, and NF‐κB were downregulated in the bergapten‐treated group.^69^

### 3.3 Betulinic acid

Betulinic acid is a pentacyclic triterpenoid from *Hyptis emoryi*. Intrathecally injected Betulinic acid (2 μg) suppressed mechanical allodynia (von Frey test) induced by paclitaxel (total 8 mg/kg, 4 times) on day 15. Butulininc acid treatment inhibited spontaneous excitatory postsynaptic currents in the lumbar spinal cord.^70^

### 3.4 Borneol

Borneol is a bicyclic monoterpene from *Artemisia* (Korean name Ssuk) and natural TRPA1 antagonist. Intrathecally treated borneol (60 μg, 10 times) suppressed oxaliplatin (total 30 mg/kg, 10 times)‐induced cold (cold plate: 2℃) and mechanical (von Frey test) hyperalgesia in mice. In addition, a single intrathecal injection of borneol (15, 30 and 60 μg) was effective against mechanical hyperalgesia.^71^

### 3.5 Cinnamic acid

Cinnamic acid is one of the key phytochemicals of *Cinnamomum cassia*. Orally administered cinnamic acid (10, 20 and 40 mg/kg) attenuated oxaliplatin (6 mg/kg)‐induced cold (acetone test) and mechanical (von Frey test) allodynia in rats. In contrast, cinnamaldehyde, another key molecule of *Cinnamomum cassia*, had no effect on oxaliplatin‐induced allodynic behaviors. Cinnamic acid also inhibited oxaliplatin‐induced hyperexcitation of wide dynamic range neurons in the spinal cord responding to mechanical (brush, press and pinch) and cold (acetone drop) stimuli on the hind paw.^72^

### 3.6 Crytotanshinone

Crytotanshinone is a diterpene isolated from *Salvia miltiorrhiza* (Korean name Dansam). In the oxaliplatin (total 24 mg/kg, 10 times) mouse model, single and multiple oral administrations of crytotanshinone (30 mg/kg, single; 10 mg/kg, 14 times) increased paw licking latency on cold plate (4 ± 1℃). In addition, crytotanshinone had inhibitory activities on glioblastoma cells, LN‐229.^61^

### 3.7 Icariin

A flavonoid derived from *Epimedium brevicornum* Maxim (Korean name Umyangkwak), icariin (100 mg/kg, p.o., 8 times) potently suppressed paclitaxel (total 24 mg/kg, 3 times)‐induced mechanical allodynia in rats from days 11 to 22. On day 15, the icariin treatment lowered the number of GFAP positive cells and the production of pro‐inflammatory cytokines (IL‐1β, IL‐6 and TNF‐α) in the spinal cord, which were up‐regulated by paclitaxel. Paclitaxel‐induced phosphorylation of NF‐κB and downregulation of SIRT1 expression in the spinal cord were inhibited by the icariin treatment.^73^

### 3.8 Incarvillateine

Incarvillateine is a monoterpene alkaloid from *Incarvillea sinensis*. Intraperitoneally injected Incarvillateine (10 and 20 mg/kg) reversed the decreased mechanical withdrawal threshold by paclitaxel (total 10 mg/kg, 5 times). The incarvillateine treatment also lowered pain behaviors in inflammatory pain and nerve injury‐induced neuropathic pain models. Adenosine antagonists blocked the analgesic effects of incarvillateine on nerve injury‐induced neuropathic pain.^74^

### 3.9 Levo‐tetrahydropalmatine

Levo‐tetrahydropalmatine is an alkaloid derived from *Corydalis yanhusuo* (Korean name Hyun‐ho‐saek). A single administration of levo‐tetrahydropalmatine (1, 2 and 4 mg/kg, i.p.) dose‐dependently relieved oxaliplatin (total 30 mg/kg, 10 times)‐induced mechanical hyperalgesia (von Frey test). Interestingly, dopamine D_1_ receptor antagonist SCH23390 (0.2 mg/kg, i.p.) blocked this anti‐hyperalgesic effect of levo‐tetrahydropalmatine (4 mg/kg). In addition, repeated daily treatment with levo‐tetrahydropalmatine (4 mg/kg, 10 times) maintained the strong anti‐hyperalgesic effect without tolerance.^75^

### 3.10 Melatonin

Melatonin, a hormone in vertebrates, has been found in several plants.^76^ Chronic oxaliplatin injections (20 mg/kg, 4 times) induced heat and mechanical allodynia in rats. Single treatment with melatonin (20 mg/kg, i.p.) increased paw withdrawal latency and threshold against radiant heat and von Frey hair, respectively. The melatonin treatment suppressed oxaliplatin‐induced increase in GFAP protein levels in the spinal cord. The melatonin treatment also downregulated mRNA levels of inflammatory mediators (IL‐1β, TNF‐α, MCP‐1, and MIP‐1 α).^77^

### 3.11 Mitragynine

Mitragynine is a major alkaloid contained in kratom. In an oxaliplatin (6 mg/kg) rat model, intraperitoneal administration of mitragynine (5 and 10 mg/kg, 5 times) reversed the decreased mechanical withdrawal latency. The anti‐allodynic effects of mitragynine were blocked by α_1_‐ and α_2_‐adreneroceptor antagonists, and opioid antagonist.^78^

### 3.12 Neoline

Neoline is an alkaloid found in Aconiti Tuber. In an oxaliplatin (10 mg/kg) mouse model, multiple administrations of neoline (10 mg/kg, s.c., 8 times) suppressed mechanical allodynia and hyperalgesia (von Frey test),^47^, ^48^ whereas attenuation of cold hyperalgesia (acetone test) was only observed in eight time neoline‐treated experiments.^47^ Neoline was detected in the rat plasma within 1 h following oral administration of Aconiti Tuber.^48^

### 3.13 *N*‐Palmitoylethanolamine

*N*‐Palmitoylethanolamine (PEA) is the endogenous amide between palmitic acid (from palm trees) and ethanolamine (from pea, oat and wheat).^79^, ^80^ Repeated daily treatment with PEA (30 mg/kg, i.p., 20 times) showed very potent analgesic effects on cold (cold plate: 4 ± 1℃) and mechanical (von Frey test) allodynia, and mechanical hyperalgesia (Randall‐Selitto test) induced by chronic injections of oxaliplatin (total 36 mg/kg, 15 times) in rats. On day 21, the PEA treatment downregulated the activation of spinal and brain astrocytes, and brain microglia induced by oxaliplatin.^81^

### 3.14 Paeoniflorin

Paeoniflorin is a monoterpene glycoside derived from Paeoniae Radix (Korean name Jak Yak). In a paclitaxel (5 mg/kg) mouse model, topical application of paeoniflorin ethanol mixture (0.1 and 1%, twice daily for 14 days) to the hind paw suppressed mechanical allodynia (0.69 mN of von Frey). The analgesic effects of paeoniflorin are inhibited by an adenosine A_1_ receptor antagonist.^82^

### 3.15 Physalin F

Physalin F is a steroid from *Physalis angulate* L. (Korean name Ttang‐kkwa‐ri). Intrathecally injected Physalin F (2 μg) suppressed paclitaxel (total 8 mg/kg, 4 times)‐induced mechanical allodynia (von Frey test) until 5 h after injection. The frequency of spontaneous excitatory postsynaptic currents in the spinal cord is inhibited by Physalin F.^83^

### 3.16 Puerarin

Puerarin is an isoflavonoid from the kudzu root (Korean name Galgeun). Local application of puerarin (1 and 10 μM) onto the dorsal roog ganglia attenuated paclitaxel (total 24 mg/kg, 3 times)‐induced mechanical allodynia (von Frey test) and thermal hyperalgesia (Hargreaves test) in rats.^84^ In another time schedule of paclitaxel treatments (total 8 mg/kg, 4 times), single administration of puerarin (20 and 40 mg/kg) showed efficacy on pre‐established mechanical allodynia and thermal hyperalgesia in rats. Moreover, repeated daily treatment with puerarin (20 mg/kg, i.p., 21 times) prevented the development of mechanical (von Frey test) and thermal (hot plate test: 53 ± 1℃) hypersensitivity. Puerarin blocked the sodium (Na_v_1.8) channels and downregulated TRPV1, CGRP and substance P in the dorsal root ganglia.^85^

### 3.17 Quercetin

Quercetin is a polyphenolic flavonoid found in various plants. Intraperitoneal treatment of quercetin (20 and 60 mg/kg) in mice (12 times) and rats (40 times) reduced mechanical allodynia (von Frey test) and heat hyperalgesia (rat: 52℃, mice 49.5℃) induced by paclitaxel (total 8 mg/kg, 4 times). Quercetin treatment lowered the PKCε and TRPV1 levels in the spinal cord.^86^

### 3.18 Rosmarinic acid

Rosmarinic acid, a natural phenolic compound from *Rosmarinus officinalis*, was orally administered (25 and 50 mg/kg, 28 times) in rats. In both doses of rosmarinic acid, oxaliplatin (total 36 mg/kg, 15 times)‐induced cold allodynia (acetone test) and hyperalgesia (cold plate: 4 ± 1℃), and mechanical hyperalgesia and allodynia were reversed. In addition, oxaliplatin‐induced activation of astrocytes and upregulation of proinflammatory cytokines (IL‐6 and TNF‐α) in the spinal dorsal horn was inhibited by rosmarinic acid (50 mg/kg).^87^

### 3.19 Saponin

Saponin is a triterpene from *Tribulus terrestris* (Korean name Nam‐ga‐sae). Vincristine (total 1 mg/kg, 10 times)‐induced mechanical allodynia (von Frey test) and hyperalgesia (Randall‐Sellitto test) were attenuated by multiple administrations of saponin (25, 50 and 100 mg/kg, p.o., 21 times). Saponin also attenuated IL‐1β, IL‐6, and TNF‐α levels in the sciatic nerve and brain as well as glutamate and aspartate levels in the brain.^88^

### 3.20 *Tabernaemontana catharinensis* ethyl acetate fraction

*Tabernaemontana catharinensis* ethyl acetate fraction (100 mg/kg, p.o.) suppressed mechanical allodynia (von Frey test) induced by a single (1 mg/kg) and multiple (total 4 mg/kg, 4 times) injections of paclitaxel in mice. The efficacy of *Tabernaemontana catharinensis* ethyl acetate fraction was mediated by the spinal TRPA1 channels.^89^

### 3.21 Tanshinone IIA

*Salvia miltiorrhiza* (Korean name Dansam) contains tanshinone IIA, a diterpene. In an oxaliplatin mouse model (total 24 mg/kg, 10 times), the decreased licking latency on cold plate was reversed in the tanshinone IIA (TIIA, 10 mg/kg, 14 times) treated group. TIIA also inhibited the activities of glioblastoma cells, LN‐229.^61^ TIIA (25 mg/kg, 10 times) treatments in an oxaliplatin rat model (total 140 mg/kg, 7 times) upregulated the decreased mechanical withdrawal threshold.^90^

## 4 CONCLUSIONS AND PERSPECTIVES

**FIGURE 1:**
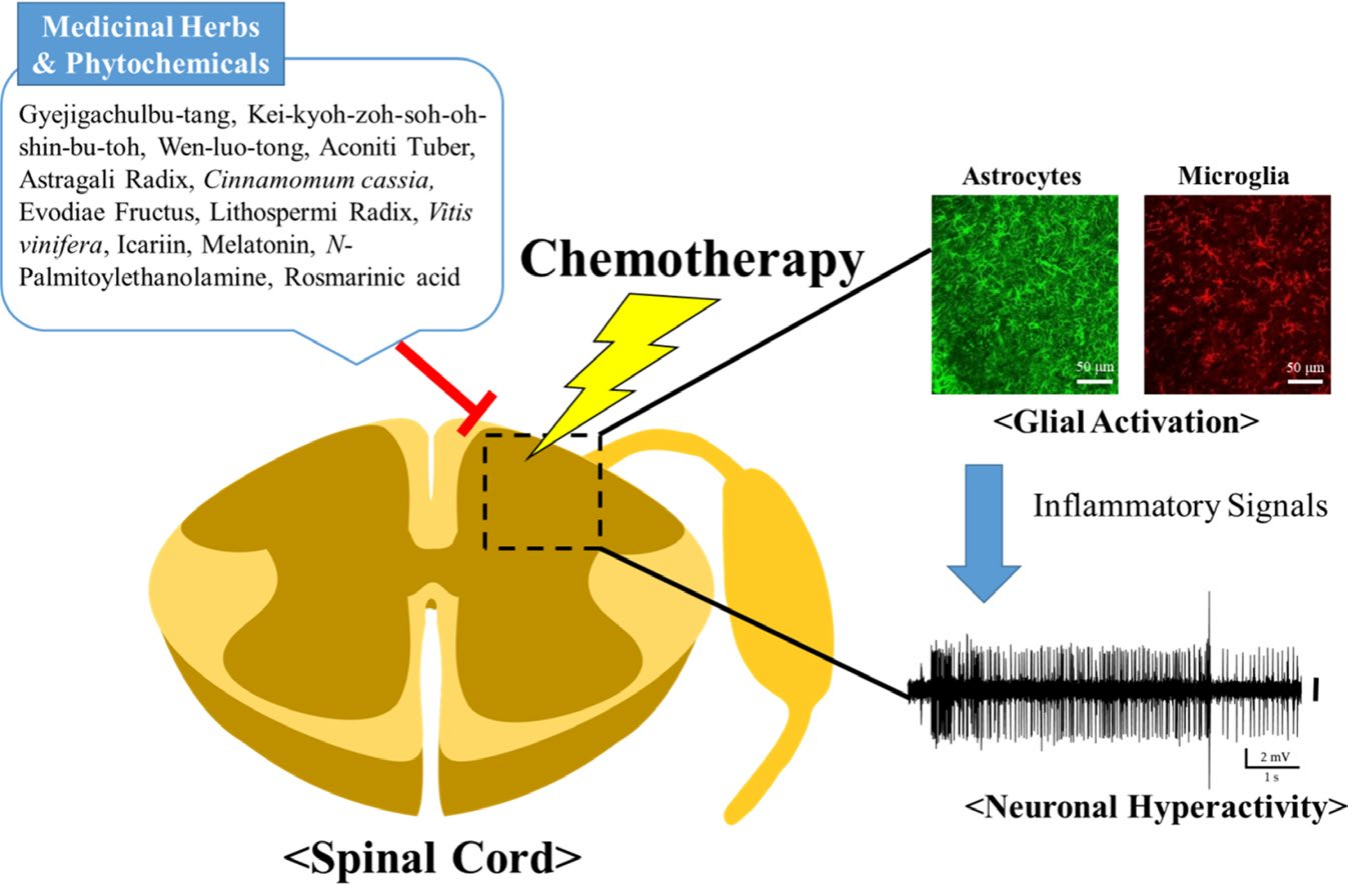
Graphical summary of this review

In this review, the use of medicinal herbs and their phytochemicals for the treatment of CIPN is summarized, focusing on glial modulation in animal models. Analgesic effects on CIPN were observed after treatment with six medicinal herb formulas, 21 single medicinal herbs, and 21 phytochemicals. Medicinal herbs or their phytochemicals are applied through various routes, such as orally, intraperitoneally, topically, or intrathecally. Both single and multiple administrations showed analgesic effects, and pretreatment also had prophylactic efficacy. Among the reviewed articles, analgesic effects accompanying glial modulation were reported in three medicinal herb formulas, six single medicinal herbs, and four phytochemicals (Table 1). A schematic diagram of this review is presented in Figure 1.

The research reviewed in this article offers high potential for the development of new drugs based on medicinal herbs. As a promising source of phytochemicals, medicinal herbs could be potent candidates for new drugs. However, it is still unclear whether medicinal herbs directly or indirectly modulate the glial activity in CIPN conditions, although there have been several reports showing the down‐regulation of oxaliplatin‐induced oxidation (O_2_^−^ production and lipid peroxidation level) in primary culture of astrocytes by hydroalcoholic extract of medicinal herbs.^67^, ^91^ Therefore, future studies are required to provide more detailed therapeutic mechanisms supporting new drug development based on medicinal herbs. How can medicinal herbs or phytochemicals act on glia in CIPN conditions? How does altered glial activity by medicinal herbs or phytochemicals affect adjacent neurons? What happens in the CNS neurons processing pain signals after treatment with medicinal herbs and phytochemicals? Answering these questions can provide information to improve their efficacies and modify their structures to better treat these conditions and others.

## DISCLOSURE

The authors declare no conflict of interest.

## AUTHOR CONTRIBUTIONS

J. H. L. and S. K. K. conceived and designed the review article; J. H. L., N. K. and S. P. wrote the manuscript draft; K. S. K. revised the manuscript.

## Data Availability

Data sharing is not applicable to this review paper, because there are no new data in this article.
